# GenAI Outperforms Human Reviewers in Classifying Alzheimer’s Disease and Related Dementia Research

**DOI:** 10.1093/geroni/igaf122.2250

**Published:** 2025-12-31

**Authors:** Duo Wei, Riya Goyal, Tasnim Raisa, Jeannine Elmasri, Jessica Fleck

**Affiliations:** Stockton University, Galloway, New Jersey, United States; Stockton University, Galloway, New Jersey, United States; Stockton University, Galloway, New Jersey, United States; Stockton University, Galloway, New Jersey, United States; Stockton University, Galloway, New Jersey, United States

## Abstract

Categorizing research literature is critical in aging-related studies [1], particularly for Alzheimer’s disease and related dementias (ADRD), where articles are typically classified into screening, diagnosis, and intervention [2]. This study compares the performance of human reviewers and GenAI in classifying ADRD literature. The human group included three trained individuals with computer science backgrounds and expertise in systematic reviews on mental health for older adults, while the AI group comprised DeepSeek (AI1), ChatGPT (AI2), and Google Gemini (AI3). Sixty-six PubMed papers were analyzed to evaluate congruence and accuracy. Congruence was measured using mutual information, a metric from information theory that quantifies shared information between variables. Results revealed strong agreement among AI models, with scores of 0.910 for AI1 and AI2, 0.686 for AI2 and AI3, and 0.776 for AI1 and AI3. In contrast, human congruency scores averaged 0.45 or below (p < 0.05). Accuracy, assessed using a majority vote and domain expert review as the gold standard, showed that human reviewers achieved an average accuracy of 0.495, while AI models performed with average of 0.757, with AI1 achieving the highest accuracy at 0.818. These findings highlight AI’s superior classification performance over human reviewers (p = 0.0019). However, challenges such as AI interpretability must be addressed. Future research should explore integrating human expertise with AI to improve the classification of ADRD literature. This study underscores the potential of AI in advancing aging-related research while emphasizing the need for careful consideration of its limitations.

